# Human cerebral organoids: cellular composition and subcellular morphological features

**DOI:** 10.3389/fncel.2024.1406839

**Published:** 2024-06-12

**Authors:** Patricia Mateos-Martínez, Raquel Coronel, Martin Sachse, Rosa González-Sastre, Laura Maeso, Maria Josefa Rodriguez, María C. Terrón, Victoria López-Alonso, Isabel Liste

**Affiliations:** ^1^Unidad de Regeneración Neural, Unidad Funcional de Investigación de Enfermedades Crónicas (UFIEC), Instituto de Salud Carlos III (ISCIII), Madrid, Spain; ^2^Unidad de Biología Computacional, Unidad Funcional de Investigación de Enfermedades Crónicas (UFIEC), Instituto de Salud Carlos III (ISCIII), Madrid, Spain; ^3^Escuela Internacional de Doctorado de la Universidad Nacional de Educación a Distancia (UNED), Programa en Ciencias Biomédicas y Salud Pública, Madrid, Spain; ^4^Unidad de Microscopía Electrónica, Unidades Centrales Científico Técnicas, Instituto de Salud Carlos III, Madrid, Spain

**Keywords:** human brain organoids, mini-brains, human pluripotent stem cells, ultrastructural characterization, transmission electron microscopy, neurodevelopment, neural stem cells, glial cells

## Abstract

**Introduction:**

Human cerebral organoids (hCOs) derived from pluripotent stem cells are very promising for the study of neurodevelopment and the investigation of the healthy or diseased brain. To help establish hCOs as a powerful research model, it is essential to perform the morphological characterization of their cellular components in depth.

**Methods:**

In this study, we analyzed the cell types consisting of hCOs after culturing for 45 days using immunofluorescence and reverse transcriptase qualitative polymerase chain reaction (RT-qPCR) assays. We also analyzed their subcellular morphological characteristics by transmission electron microscopy (TEM).

**Results:**

Our results show the development of proliferative zones to be remarkably similar to those found in human brain development with cells having a polarized structure surrounding a central cavity with tight junctions and cilia. In addition, we describe the presence of immature and mature migrating neurons, astrocytes, oligodendrocyte precursor cells, and microglia-like cells.

**Discussion:**

The ultrastructural characterization presented in this study provides valuable information on the structural development and morphology of the hCO, and this information is of general interest for future research on the mechanisms that alter the cell structure or function of hCOs.

## 1 Introduction

The study of the human brain is challenging due to its functional and structural complexity, and the challenge in extrapolating data obtained from animal models arises from the differences between their brain and the human brain (Spires and Hyman, 2005; Benito-Kwiecinski et al., 2021; Fernandes et al., 2021). Innovative scientific advances derived from models using human pluripotent stem cells (hPSCs) represent a breakthrough in the study of the brain (Engle et al., 2018; Baldassari et al., 2020). The generation of human cerebral organoids (hCOs) (Lancaster et al., 2013) makes it possible to conceive a three-dimensional (3D) model that functionally and structurally resembles the human brain (Eichmüller and Knoblich, 2022). The hCOs offer many opportunities in the study of human neurodevelopment (Camp et al., 2015; Nascimento et al., 2019), as well as in the study of diseases and neurodegeneration of the brain (Eichmüller and Knoblich, 2022).

There are several protocols developed for the generation of hCOs (Lancaster et al., 2013; Pașca et al., 2015; Quadrato et al., 2016; Sloan et al., 2018; Trujillo et al., 2019; González-Sastre et al., 2024). The cell types present in hCOs reflect broad expression profiles of human neural cell types and can be observed in publicly available single-cell RNA sequencing (scRNAseq) datasets from multiple organoid protocols (Andrews and Kriegstein, 2022). However, many questions remain unanswered about the morphology of the cells that constitute hCOs for research use (Nahirney and Tremblay, 2021).

Electron microscopy (EM) (Bozzola, 2007; Gordon, 2014; Harris, 2015) is a powerful technique to capture the ultrastructural details that cannot be detected by other methods such as immunofluorescence imaging and scRNA sequencing. Thus, the use of transmission electron microscopy (TEM) allows for delving deeper into the functions performed by glial cells and neurons (Savage et al., 2018; Marton et al., 2019; Capetian et al., 2020; Aten et al., 2022). Most EM studies have been performed in rodent or primate models (Luskin et al., 1993; Peters and Sethares, 2004; Turegano-Lopez et al., 2022; Turner et al., 2022) or in post-mortem human brains (Cragg, 1976; Lewis et al., 2019), which do not reflect the structural complexity of the human brain during neurodevelopment.

In this study, hCOs generated following the protocol of González-Sastre et al. (2024) were characterized at a nanometer resolution after culturing for 45 days. The ultrastructure of the neuroepithelial zones, neurons, astrocytes, oligodendrocyte precursors, and microglia-like cells were observed.

## 2 Materials and methods

### 2.1 Cell culture

The human embryonic pluripotent stem cell line (hESC) AND-2, which was obtained from the Biobanco de células madre de Granada (ISCIII, Spain), was used for this study. The study was approved by the ISCIII Ethics Committee (Ref. CEI-PI-93_2020 and CEI-PI-76_2023) and then authorized by the Spanish National Committee of Guarantees for the Use and Derivation of Human Cells and Tissues.

The AND-2 cell line was grown on mouse embryonic fibroblasts (MEFs) treated and inactivated overnight with Mitomycin C (1 μg/ml, Sigma, M0503). The culture was maintained in an hESC proliferation medium. This medium consists of knockOut DMEM (Gibco, 10829-018) supplemented with serum replacement (20%, Gibco, 10829-028), L-glutamine (2 mM, Lonza, BE17-605E), non-essential amino acids (1X, Gibco, 11140-050), penicillin/streptomycin (50 U/ml, Lonza, DE17-602E), 2-mercaptoethanol (50 μM, Gibco, M6250), fibroblast growth factor 2 (FGF2) (10 ng/ml, PeproTech, AF-100-18B), and ROCK Inhibitor Y-27632 (5 μM, Tocris, 129830-38-2). This medium was changed with a fresh medium every day, and the culture was maintained at 37°C at 5% CO_2_.

### 2.2 Generation of human cerebral organoids (hCOs)

To obtain human cerebral organoids, we relied on the protocol previously described by González-Sastre et al. (2024).

After obtaining an optimal density of AND-2 colonies, the hESC proliferation medium was changed to a neural induction medium. The neural induction medium consists of N2 [DMEM: F12 with GlutaMax (Gibco, 31331-028) with D-glucose (6 mg/ml, Merck, 104074), N2 supplement (1X, Gibco, 17502-048), AlbuMAX (2.6 mg/ml, Gibco, 11020-021), penicillin/streptomycin (50 U/ml, Lonza, DE17-602E), HEPES (5 mM, Gibco, 15630-056), non-essential amino acids (1X, Gibco, 11140-050)], Neurobasal without vitamin A [Neurobasal (Gibco 21103-049) with B27 supplement without vitamin A (1X, Gibco, 12587-010), L-glutamine (2 mM, Lonza, BE17-605E)] at a ratio of 1:1 supplemented with L-ascorbic acid (0.1 mM, Sigma, A4544), SB-431542 (10 μM, Tocris, 301836-41-9), Noggin (50 ng/ml, PeproTech, 120-10C), and CHIR99021 (3 μM, Tocris, 252917-06-9).

After 5–7 days, the colonies began to enlarge, and three-dimensional (3D) structures began to detach, which were transferred to a new multiwell plate pretreated with an anti-adherent solution (StemCell Technologies, 7010).

Subsequently, 5–7 days after the formation of the 3D structures, the neural induction medium was replaced by a neural differentiation medium. This medium consists of N2 and Neurobasal without vitamin A in a 1:1 ratio supplemented with 1.3 μg/ml of insulin (Gibco, 12585-014).

After another 5–7 days with a neural differentiation medium, it was replaced by a maintenance medium. This medium consists of N2 and Neurobasal with vitamin A [Neurobasal with B27 supplement with vitamin A (1X, Gibco, 17504-044) and 2mM L-glutamine] in a 1:1 ratio supplemented with 1.3 μg/ml of insulin.

During the whole process, the medium corresponding to each phase was replaced by a fresh medium every day. They were incubated at 37°C with 5% CO_2_.

### 2.3 Immunohistochemistry (IHC)

Once the organoids reached the desired maturity, after 45 days of culturing, they were fixed using Paraformaldehyde (PFA, 4%, Sigma, P6148) and embedded in gelatin (7.5%, Merck, 104070) and sucrose (15%, Merck, 107654). Sections of 15 μm were made using a cryostat (Leica).

These sections were blocked and permeabilized (1 h at room temperature (RT) in a blocking solution [Triton X-100 (0.3%, BioRad, 161-0407), normal horse serum (5%, NHS; Gibco, 2493028), and bovine serum albumin (BSA) (0.1%, Sigma, A7906) in PBS)] for immunohistochemical analysis. Sections were incubated with primary antibodies diluted in the blocking solution overnight at 4°C: rabbit anti-Ki67 [1:250] (Thermo Scientific, MA5-14520), mouse anti-Vimentin [1:500] (Santa Cruz, SC-6260), rabbit anti-Sox2 [1:1000] (Millipore, AB5603), mouse anti-ZO1 [1:200] (Invitrogen, 33-9100), mouse anti-βIIITubulin [1:500] (Biolegend, 801202), goat anti-DCX [1:100] (Santa Cruz, SC-8066), mouse anti-MAP2 [1:200] (Sigma, M4403), rabbit anti-SYN1 [1:200] (Millipore, AB1543), rabbit anti-S100β [1:100] (Abcam, ab52642), rabbit anti-GFAP [1:800] (Dako, Z0334), mouse anti-CNPase [1:150] (Millipore, MAB326), and rabbit anti-IBA1 [1:400] (Wako, 019-19741).

After the removal and washing (0.25% Triton X-100 in PBS) of the primary antibody, the sectioned organoids were incubated with the corresponding secondary antibody (for 1 h at RT): donkey anti-rabbit Alexa Fluor 448 [1:500] (Invitrogen, A21206), donkey anti-mouse Alexa Fluor 555 [1:400] (Invitrogen, A31570), donkey anti-goat Alexa Fluor 555 [1:400] (Invitrogen, A21432), and donkey anti-rabbit Alexa Fluor 555 [1:400] (Invitrogen, A31572). Finally, the nuclei were stained with Höechst 33258 (Thermo Fisher) diluted in PBS (1:500) (for 5 min at RT).

A Leica DMi8 fluorescence microscope was used for section analysis and photography. Photographs were taken at 10 × or 20 × magnification. All images were obtained with Leica LAS X V4.0 software.

### 2.4 RNA extraction and real-time quantitative PCR (RT-qPCR)

The RNeasy Mini extraction kit (Qiagen, 74104) was used, as indicated in the instructions, to obtain RNA from hCOs. The hCOs were treated with DNAses to avoid the amplification of non-specific genomic DNA. The reverse transcription of 1 μg of total RNA (10 min at 25°C, 60 min at 50°C, and 10 min at 75°C) was performed in a 20-μl reagent mix using SuperScriptIII-RT (Invitogen, 56575). Relative cDNA levels were quantified by RT-qPCR (PowerUp SYBR-green system, Applied Biosystems, A25742) in a 15-μl reaction, with 10 μM of primers and 10 ng of cDNA. This quantification was performed at different stages: on organoids at the induction stage (5 days), at the differentiation stage (14 days), and during maintenance (45 days).

The human genes used were as follows: *MKI67* (F: TGACCCTGATGAGAAAGCTCAA; R: CCCTGAGCAACACT GTCTTTT), *VIM* (F: TACAGGAAGCTGCTGGAAGG; R: ACCAGAGGGAG-TGAATCCAG), *SOX2* (F: GGGGGAA TGGACCTTGTATAG; R: GCAAAGCTCCTACCGTACCA), *SYN1* (F: GACGGAAGGGATCACATCAT; R: CTGGTGG TCACCAATGAGC), *DCX* (F: GGATCCAGGAAGATCGGAAG; R: TTGTCTGAGGAACAGACATAGCT), *MAP2* (F: ATCTCT TCTTCAGCACGGCG; R: CAGGGGTAGTGGGTGTTGAG), *TUBB3* (F: GCAACTACGTGGGCGACT; R: ATGGCTCGA GGCACGTACT), *S100B* (F: GGAAGGGGTGAGACAAGGA; R: GGTGGAAAACGTCGATGAG), *GFAP* (F: GTTCTT GAGGAA-GATCCACGA; R: CTTGGCCACGTCAAGCTC), *AIF1* (F: TTAATGGAAATGGCGATATTGA; R: TTCTTTAGCTCTAGG TGAGTCTTGG), *PLP1* (F: GGCTAGGACATCCCGACAAGTT; R: ACAGCAGAGCAGGCAAACAC), *PDGFRA* (F: TACACTT GCTATTACAACCACA; R: ATCCTCCACGATGACTAAAT), and housekeeping gene *TBP* (F: GAGCTGTGATGTGAAGTTTCC; R: TCTGGGTTTGATCATTCTGTAG).

RT-qPCR from Applied Biosystems QuantStudio 3 was used to determine the levels of target mRNA in each sample, normalizing the relative expression levels against the *TBP* levels of each sample. The relative gene levels were estimated by relative quantification using the 2^−ΔΔ*Ct*^ method.

### 2.5 Statistical analysis

Statistical analyses were performed using GraphPad Prism 9. The results are shown as mean ± SD for three experiments (*n* = 3). Data were compared by a multiple comparison ANOVA. The *p* < 0.05 were considered to be statistically significant (^*^p < 0.05; ^**^p < 0.01; ^***^p < 0.001; ^****^p < 0.0001).

### 2.6 Preparation of cerebral organoids (hCOs) for electron microscopy

Once the organoids were cultured for ~45 days, they were collected in a sodium phosphate buffer (0.1M, 7.4 pH) and fixed in the same buffer with 2% glutaraldehyde and 4% PFA for 2 h at room temperature (RT).

The hCOs were washed with sodium phosphate buffer three times. Post-fixation was done in the following order: 1% osmium tetroxide and 1% potassium ferricyanide (for 1 h at 4°C), 0.15% tannic acid (for 1 min at RT), and 2% uranyl acetate (for 1 h at RT in the dark).

Samples were dehydrated (10 min, 4°C) in ethanol with increasing concentrations (50%, 75%, 90%, 95%) and, finally, 100% ethanol (three times, 12 min, 4°C) and infiltrated with increasing concentrations of epoxy-resin (50%, 75%, 100%) before polymerization at 60°C for 48 h.

Ultrathin sections of 70 nm were cut using a Leica EM UC6 ultramicrotome. Images were obtained using a Talos F200CG2 transmission electron microscope equipped with a BM-CETA camera. Images were analyzed using MAPS 3.23 software.

### 2.7 Single-cell RNA-seq of cerebral organoids (hcos)

The hCOs were collected with the aid of a pipette and treated for 15 min in Hank's Balanced Salt Solution (HBSS; 1X, Gibco, #14180046). After centrifugation, the dissociation was performed with trypsin for 20 min at 37°C and with DMEM:F12 + 10% FBS in a 1:1 ratio. Counting and viability were assessed using Trypan blue staining. The single-cell library was constructed using the Chromium Single Cell 3′ Library and Gel Bead Kit v3.1 workflow from 10 × Genomics at the Genomics Unit of the Instituto de Salud Carlos III, and it was sequenced using the Novaseq 6000Dx sequencer (Illumina). After using CellRanger Count (version 6.1.2. 10X Genomics), data analysis was performed using the Seurat (v4.0). Data visualization was carried out using the Uniform Manifold Approximation and Projection (UMAP), and the marker-based cell and cluster assignment were performed according to González-Sastre et al.'s (2024) protocol. Transcriptomic scRNAseq data can be retrieved using Gene Expression Omnibus (GEO) series accession number GSE266667 in NCBI.

We used the R packages Harmony within our Seurat workflow to perform the integration of the scRNAseq data and MAST to achieve the differential gene expression analysis. The summarized gene ontology terms were obtained from Enrichr (https://maayanlab.cloud/Enrichr/) and ReviGO (http://revigo.irb.hr/).

## 3 Results

### 3.1 Generation of the human cerebral organoids and characterization of proliferative zones

**Figure 1:**
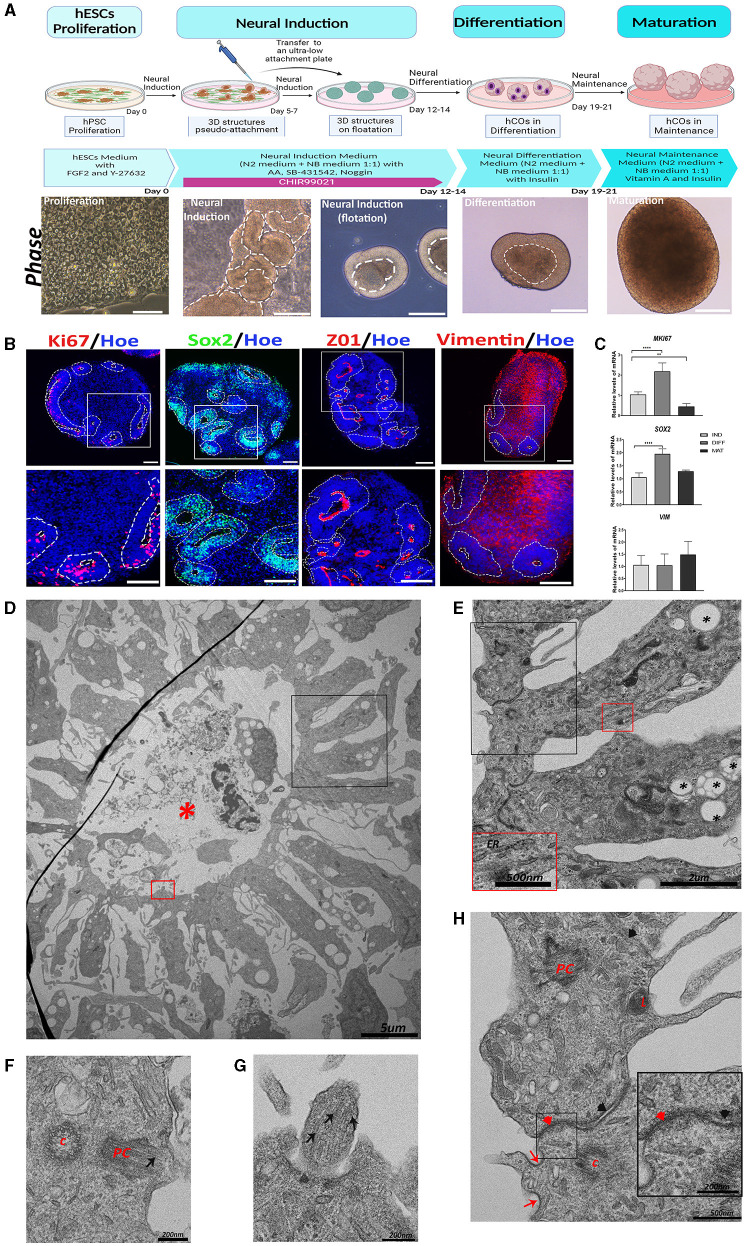
Generation of human cerebral organoids (hCOs) directly from adherent cultures of pluripotent stem cells. Proliferative zone in the hCOs. **(A)** A schematic representation of the timeline of the protocol employed to generate the hCOs. Bright-field images of the morphological changes associated with each stage are presented below each phase of the protocol. White dashed lines mark the structures corresponding to the neuroepithelium. Scale bar = 100 μm (proliferation), 200 μm (neural induction, differentiation, and maturation). **(B)** Representative images of the hCOs immunostained for Sox2 (green), Ki67, and Vimentin (red) to show the proliferative cells, marked with white dashed lines, and ZO1 (red), a tight junction marker. Nuclei are stained with Höechst (blue). Scale bar = 100 μm. **(C)** Relative quantification of gene expressions of *SOX2, VIM*, and *MKI67* by RT-qPCR. Data represent mean ± SD (*n* = 4 for each experiment). Statistical analysis was performed using a one-way ANOVA. ^**^p < 0.01; ^****^p < 0.0001. **(D)** Representative transmission electron microscopy (TEM) image of a proliferative zone with debris and cells in the central cavity (red asterisk). Scale bar = 5 μm. **(E)** The magnified black boxed area in image **(D)** shows the cells that conform to the proliferative zone. These cells contain abundant lipid vesicles (black asterisk) and endoplasmic reticulum (ER). Scale bar = 2 μm. In the magnification (red box magnification, scale bar = 500nm), the ER is shown in more detail. **(F)** TEM image of a cell of the proliferative zone that shows a centriole (c) and a primary cilium (PC). The microtubules (black arrow) of the primary cilium are visible. Scale bar = 200 nm. **(G)** The magnified red-boxed area in image **(D)** shows part of a primary cilium with the microtubules (black arrow). Scale bar = 200 nm. **(H)** Magnification of the black boxed area of image **(E)** showing the organelles and cellular connections. Inside these cells, the following can be observed: lysosomes (L), primary cilium (PC) in development, and centriole **(C)**. In the apical region between the two cells, tight junctions (red arrowhead) and clathrin-coated pits (red arrow) are observed; while in the distal region, adherent junctions (black arrowhead) are visible. Scale bar = 500 nm. In the magnification (black box, scale bar = 200 nm), these tight and adherent junctions can be seen in more detail. ZO1, Zonula Occludens-1; *VIM*, Vimentin; IND, neural induction; DIFF, differentiation; MAT, maturation.

To obtain hCOs from hPSCs, we used the recently described protocol from our group (González-Sastre et al., 2024), which allows the generation of hCOs directly from the adherent cultures of pluripotent stem cells (PSCs) avoiding embryoid body formation in the detachment and aggregation stages. The different phases of proliferation, neural induction, differentiation, and maturation with the corresponding timelines and representative bright-field images of the stages are shown in Figure 1A.

The cellular and molecular characterization of the generated hCOs was performed after culturing for 45 days using immunohistochemistry and RT-qPCR for several markers. As can be observed in Figure 1B, hCOs presented an abundant number of proliferative zones (white dashed lines), with high immunoreactivity for Ki67 (a cell proliferation marker), Sox2 (a marker of neural precursor cells), ZO1 (Zonula Occludens-1, a marker of tight junction proteins) (Aaku-Saraste et al., 1996), and Vimentin (an intermediate filament protein found in many types of immature cells throughout the central nervous system (CNS), including radial glial cells (RGCs) and immature astrocytes) (Hohmann and Dehghani, 2019).

The gene expressions of *SOX2* and *MKI67* were higher at the differentiation stage but decreased at the maturation stage, as hCOs differentiate and maturate (Figure 1C). Nevertheless, *VIM* expression remains constant, as would be expected given that it is present in a variety of cell types at different stages of differentiation.

To further characterize the hCOs morphologically, we used transmission electron microscopy (TEM) to observe a representative image of a proliferative zone with a central cavity containing cell debris and intact cells, as shown in Figure 1D, in parallel cultures of 45 days. This cavity is surrounded by radially oriented cells that are connected to each other by tight junctions in the apical region, sometimes with a clathrin-coated pit near them, and by adherent junctions in the distal region (Figures 1E, H). These cells contain rough endoplasmic reticulum and abundant lipid vesicles (Figure 1E). In addition, in some of these cells, a centriole and a primary cilium composed of microtubules could be observed (Figures 1F–H).

These proliferative zones are of significant importance for the correct development and differentiation of neurons, astrocytes, and oligodendrocytes (Conti and Cattaneo, 2010). It has been previously observed in several protocols of hCOs how the aforementioned cell types are generated, differentiated, and migrated radially outward from these proliferative zones, which are composed of neural precursor cells (Lancaster and Knoblich, 2014; Pașca et al., 2015). In addition, neurodevelopmental changes have been observed when these regions are altered (Camblor-Perujo and Konon-enko, 2022; Bear and Caspary, 2024).

### 3.2 Neuronal differentiation in the hCOs

To verify the existence of differentiated and mature neurons in hCOs, we analyzed the expression of several neuronal markers. Doublecortin (DCX), a microtubule-stabilizing protein essential for neuronal migration during human brain development, is a marker of migrating neuroblasts. βIIITubulin (BIIITub), a microtubule element of the tubulin family found almost exclusively in neurons, is a marker of differentiated neurons. Microtubule-Associated Protein 2 (MAP2), the predominant cytoskeletal regulator within neuronal dendrites, is a marker of mature neurons. Synapsin-1 (SYN1), a protein present in pre-synaptic terminals, is a marker of synapsis.

**Figure 2:**
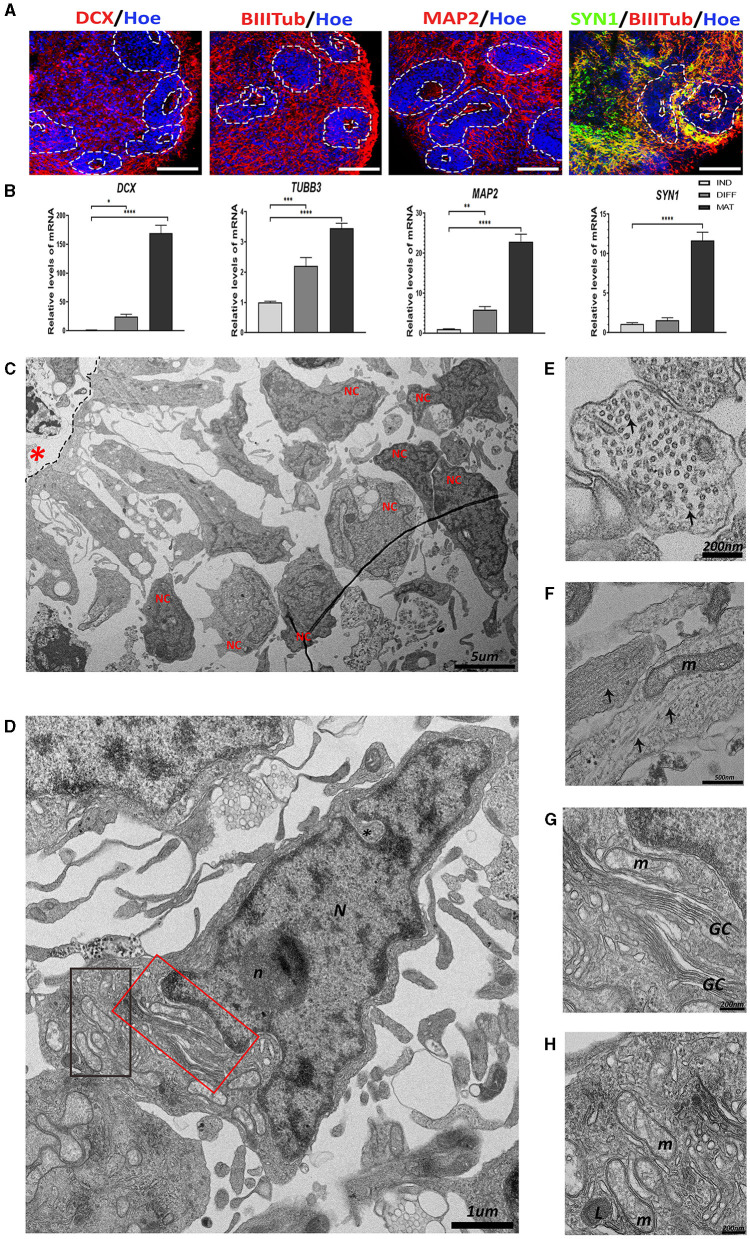
The presence of neurons in the human cerebral organoids (hCOs). **(A)** Representative images of the hCOs immunostained for DCX, BIIITub, MAP2 (red), SYN1 (green), and Höechst (blue). White dashed lines represent the area corresponding to the proliferative zones. Scale bar= 100 μm. **(B)** Relative quantification by RT-qPCR of *DCX, TUBB3, MAP2*, and *SYN1*. Data represent mean ± SD (*n* = 4 for each experiment). Statistical analysis was performed using a one-way ANOVA. *p < 0.05, **p < 0.01; ***p < 0.001; ****p < 0.0001. **(C)** Overview of the transmission electron microscopy (TEM) image of the neurons (NC) differentiated from the proliferative zone (black dashed lines) in the hCOs. Scale bar = 5 μm. **(D)** TEM image of a neuron. The nucleus (N) has some indentation in the nuclear membrane (black asterisk) and a defined and rounded nucleolus (n). The cytoplasm has different organelles. Scale bar = 1 μm. **(E)** Cross-section of a neurite. Microtubules (two highlighted by black arrows) are arranged inside. Scale bar = 200 nm. **(F)** Longitudinal section of neurites. The microtubules (black arrows) can be seen arranged in parallel to each other, next to a mitochondrium (m). Scale bar = 500 nm. **(G)** The magnified red boxed area in image **(D)** showing the Golgi complex (GC). Scale bar = 200 nm. **(H)** The magnified black boxed area in image **(D)** showing mitochondria (m) and lysosome (L). Scale bar = 200 nm. Red asterisk: central cavity of rosette; DCX, Doublecortin, BIIITub/*TUBB3*, βIIITubulin; MAP2, Microtubule-Associated Protein 2; SYN1, Synapsin-1; IND, neural induction DIFF differentiation; MAT, maturation.

As shown in Figure 2A, abundant immunoreactivity for DCX, BIIITub, MAP2, and SYN1 was observed in hCOs. These results were confirmed at the gene expression level using RT-qPCR, where a significant increase in the expression of all these genes was obtained at the maturation stage of hCOs (Figure 2B). All these data suggest the presence of migrating immature and mature neurons in the hCOs.

The TEM image of a representative proliferative zone (Figure 2C) shows the migration of radial-glia-like cells from the apical region and the differentiation into neuron-like cells in the distal region. The morphology of a representative neuron is presented in Figure 2D. Neurons exhibit a large nucleus, with indentations, a narrow ring of high electron density of heterochromatin, and one electron-dense round nucleolus. In addition, abundant organelles can be observed such as the extensive Golgi complex and mitochondria (Figures 2D, G, H). Interestingly, neurites (the neuron prolongations) contain an extensive array of microtubules that can be observed in transversally sectioned neurites (Figure 2E) or longitudinally sectioned neurites (Figure 2F), together with mitochondria.

### 3.3 The presence of astrocytes in the hCOs

**Figure 3:**
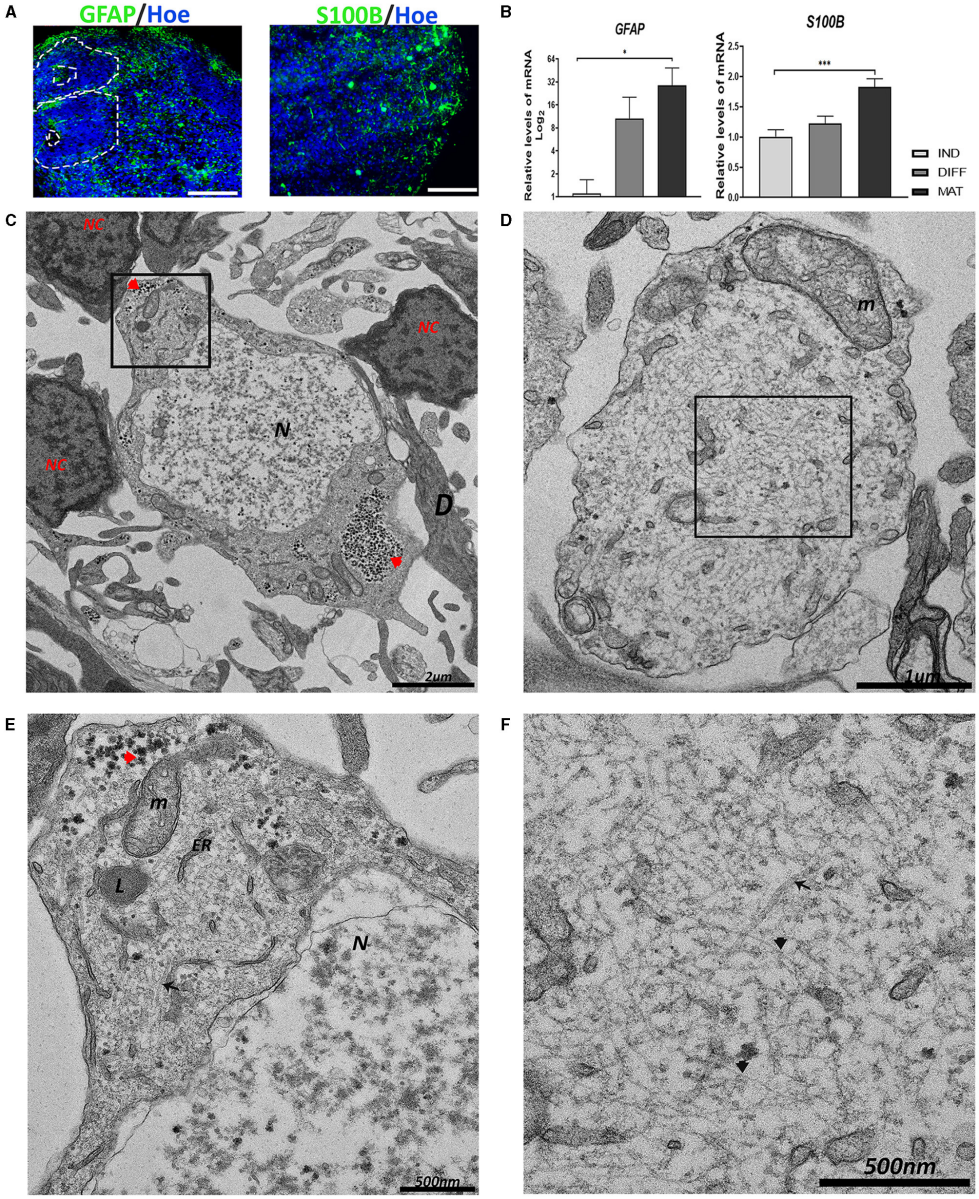
Generation of astrocytes in the hCOs. **(A)** Representative images of the hCOs immunostained for GFAP, S100β (green), and Höechst (blue). White dashed lines represent the area corresponding to the proliferative zones. Scale bar = 100 μm. **(B)** Relative quantification by RT-qPCR of *GFAP* and *S100B*. Data represent mean ± SD (*n* = 4 for each experiment). Statistical analysis was performed using a one-way ANOVA. *p < 0.05; ***p < 0.001. **(C, D)** Representative transmission electron microscopy (TEM) images of astrocytes. The astrocyte in image **(C)** is surrounded by several neurons (NC) and dendrites (D). The nucleus (N) shows euchromatin. Glycogen inclusions could be seen in the cytoplasm (red arrowhead). Scale bar = 2 μm. The astrocyte in image **(D)** shows an astrocyte process with a big mitochondrion (m). Scale bar = 1 μm. **(E)** The magnification of the black boxed area of image **(C)**. The cytoplasm contains glycogen inclusions (red arrowhead), together with numerous mitochondria (m), endoplasmic reticulum (ER), and lysosomes (L). Scale bar = 500 nm. **(F)** The magnification of the black boxed area of image **(D)**. The multitude of intermediate filaments (black arrowhead) expanding in the cytoplasm is visible, and some scattered microtubules (black arrow). Scale bar = 500 nm. GFAP, Glial Fibrillary Acidic Protein; IND, neural induction; DIFF, differentiation; MAT, maturation.

Apart from neurons, other cell types (astrocytes and oligodendrocytes) develop from the proliferative zones at the later stages of neurogenesis (Arellano et al., 2021). As in other hCO generation protocols, the presence of astrocytes has been described (Lancaster et al., 2013; Pașca et al., 2015). Given their importance in brain function (Guillamón-Vivancos et al., 2015), we aimed to study the existence of astrocytes in the hCOs. Therefore, we analyzed the expression of two markers: glial fibrillary acid protein (GFAP), which is an intermediate filament protein expressed primarily by astrocytes, and a Ca^2+^-binding protein (S100β), which is expressed and secreted from astrocytes. Immunoreactivity for both proteins is observed in hCOs, as shown in Figure 3A. The mRNA expression levels for *GFAP* and *S100B* have been significantly increasing with time in the culture (Figure 3B).

At the ultrastructural level using TEM, astrocytes appear surrounded by dendrites and neuronal-like cells, which are characterized by an irregular nucleus with a high presence of euchromatin compared with the other cell types, with more electron-translucent areas than its cytoplasm (Figures 3C, D). Astrocytes contain numerous glycogen granules, mitochondria, endoplasmic reticulum, and lysosomes (Figure 3E). In addition, astrocytes are rich in intermediate filaments (IFs) interspersed with some scattered microtubules (Figure 3F).

### 3.4 hCOs contain oligodendrocytes precursor cells (OPCs) and microglia-like cells

Other cell types that perform essential functions in the brain are oligodendrocytes and microglia cells. Therefore, we aimed to analyze their presence in hCOs.

**Figure 4:**
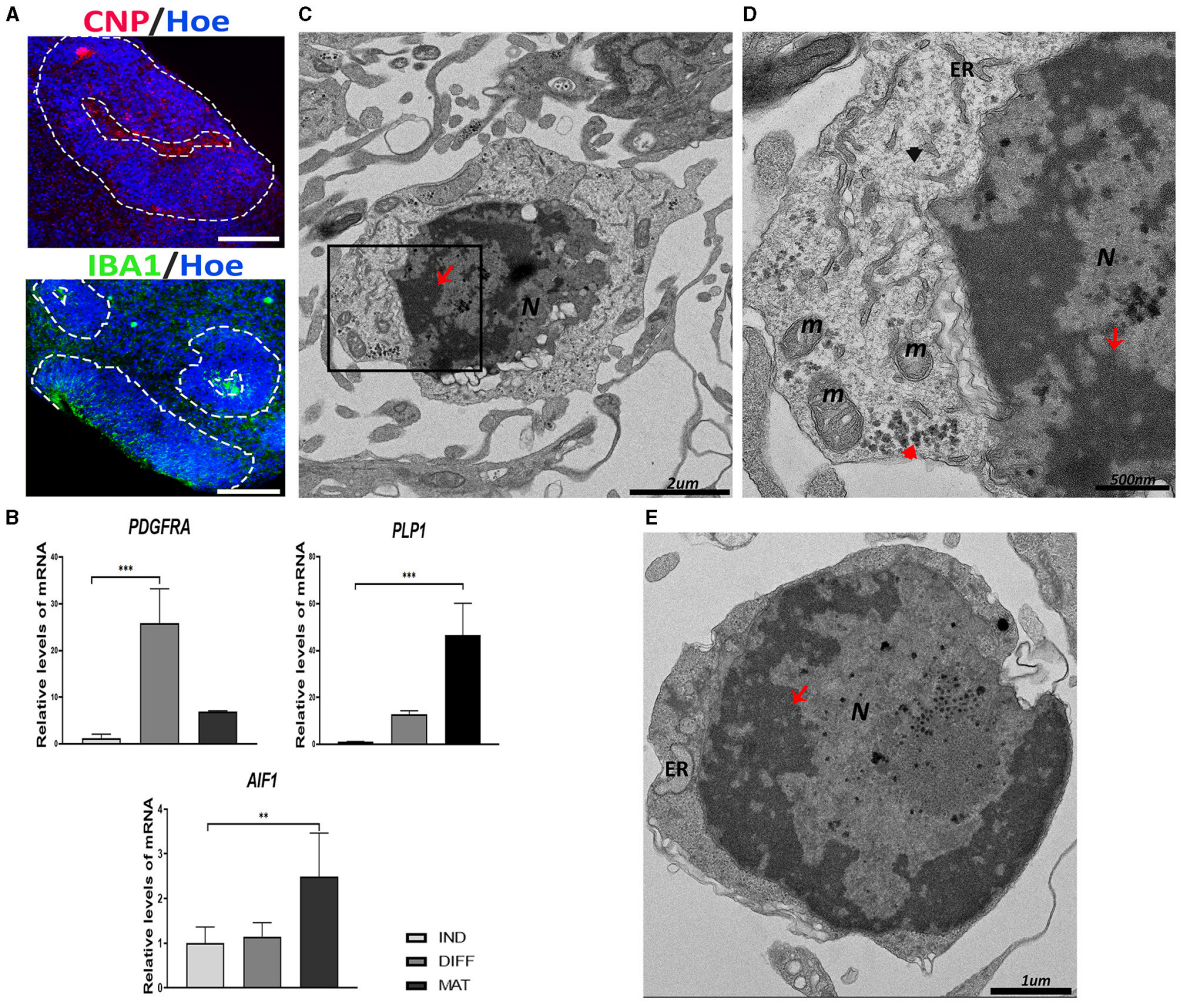
Oligodendrocyte precursor cells (OPCs) and microglial-like cells found in the cerebral organoids (hCOs). **(A)** Representative images of the hCOs immunostained for CNP (red), IBA1 (green), and Höechst (blue). White dashed lines represent the area corresponding to the proliferative zones. Scale bar = 100 μm. **(B)** Relative quantification by RT-qPCR of *PDGFRA, PLP1*, and *AIF1*. Data represent mean ± SD (*n* = 4 for each experiment). Statistical analysis was performed using one-way ANOVA. **p < 0.01; ***p < 0.001. **(C)** Representative transmission electron microscopy (TEM) images of an oligodendrocyte. The nucleus (N) contains abundant heterochromatin (red arrow). Scale bar = 2 μm. **(D)** The magnification of the black boxed area of image **(C)**. The cytoplasm contains glycogen deposits (red arrowhead), mitochondria (m), endoplasmic reticulum (ER), and intermediate filament (black arrowhead). The nucleus shows heterochromatin (red arrow). Scale bar = 500 nm. **(E)** TEM image of a microglial cell with abundant heterochromatin (red arrow) in the nucleus (N). Endoplasmic reticulum (ER) can be seen in the scant cytoplasm. Scale bar = 1 μm. IBA1/*AIF1*, ionized Ca^2+^-binding adapter protein 1; *PDGFRA*, platelet–derived growth factor receptor alpha; *PLP1*, proteolipid protein 1; CNP, CNPase; IND, neural induction; DIFF, differentiation; MAT, maturation.

To detect the existence of oligodendrocyte precursor cells (OPCs), we used immunolabeling for CNPase, the earliest myelination-specific protein produced by oligodendrocytes (Figure 4A). In addition, as shown in Figure 4B, we observed an increase in the expression of *PDGFRA*, the gene implicated in the migration of oligodendrocyte precursors, and *PLP1*, the gene encoding a transmembrane proteolipid protein that is the predominant component of myelin.

At the ultrastructural level, the OPCs have a round, electron-dense nucleus with aggregated heterochromatin (Figure 4C). The cytoplasm contains short cisternae of rough endoplasmic reticulum with numerous short mitochondria and some glycogen granules and IFs (Figure 4D).

On the other hand, the presence of microglial-like cells in hCOs obtained after culturing for 45 days was observed by immunostaining with IBA1 (ionized Ca^+2^-binding adapter protein 1), an actin-interacting protein in microglia (Figure 4A). The expression of the gene encoded for IBA1, that is, the Allograft Inflammatory Factor 1 (*AIF1*), was significantly increased in the maturation stage (Figure 4B).

The ultrastructure of a typical microglial cell is shown in Figure 4E, which is composed of a large nucleus surrounded by a thin cytoplasm. The nucleus presents a dense heterochromatin lining the nuclear membrane, contrasting with a narrow rim of perinuclear cytoplasm containing a few organelles.

### 3.5 scRNAseq of the hCOs

The progression of cell identities and possible functional changes that arise during the maturation stage of the hCOs was studied by the comparison of the transcriptional profile after culturing for 45 and 60 days [this scRNAseq data were published in a study by González-Sastre et al. (2024); GEO accession number GSE242329]. Supplementary Figures S1A–C show that, during organoid maturation, there is an increase in the proportions of cells in the hCO clusters corresponding to outer radial glia (oRG), excitatory neurons (NE), inhibitory neurons (IN), and oligodendrocyte precursor cells (OPCs), with a decrease in the proportions of cells in the clusters of apical radial glia (aRG) and intermediate precursor cells (IPCs). Supplementary Figure S1D shows that the expression of Cajal-Retzius, choroid plexus, dorsal anterior encephalon, hippocampus, and mid and posterior encephalon marker genes increases with an increase in the number of days in culture. Supplementary Figure S1E and Supplementary Table S1 show selected representative biological process GO terms enriched in the upregulated and downregulated differentially expressed genes (DEGs) identified during the comparison of hCOs after culturing for 60 days vs. after culturing for 45 days for the EN cluster.

## 4 Discussion

In the last decade, the number of publications on hCOs has increased remarkably. However, ultrastructural studies dealing with specific cellular features in these hCOs are still scarce (Chandrasekaran et al., 2017; De Kleijn et al., 2023; Pavlinov et al., 2023). In this study, we delve into the cellular structural findings that we have observed in hCOs after culturing for 45 days.

Human brain development starts from a population of neural precursors that are arranged in the shape of a rosette (Conti and Cattaneo, 2010; Andrews and Kriegstein, 2022). The ultrastructure and functional organization of these proliferative zones have been studied in adult vertebrates (Garcia-Verdugo et al., 2002), but its study during human neurodevelopment is still of great complexity. We found the formation of proliferative regions with a central cavity and highly polarized cells with pale cytoplasm and intermediate filaments (IFs) in the hCOs. In the ventral (or apical) zone of the proliferative region, some precursor cells show epithelial features such as tight junctions, while in the basal (or outer) zone, some cells show glial polarity features such as adherent junctions (Arellano et al., 2021). Some of these precursor cells present lipid vacuoles that probably are essential to maintain the apicobasal polarity by an interplay between polarity proteins and phospholipids (Chou et al., 2018).

Cells in this proliferative zone transit into radial glial cells (RGCs) to mark the onset of neurogenesis. These proliferative ventricular zones are of great interest, and most of the neurons in the brain are derived either directly or indirectly from the RGCs, in addition to their role as a scaffold for the radial migration of neurons and as precursors of astrocytes (Eze et al., 2021). One of the main characteristics of the RGCs is the loss of the tight junctions at the terminal foot (Lendahl et al., 1990; Aaku-Saraste et al., 1996), which is previously associated in vertebrates with the decrease of specific proteins such as Zonula Occludens-1 (ZO1), occludins, and claudins (Chou et al., 2018). Adherent junctions are found in the cells of the basal zone and their composition of calcium-dependent adhesion molecules, such as cadherins, are crucial to keep the apical-basal polarity and to prevent their premature differentiation into neurons (Miranda-Negron and Garcia-Arraras, 2022). This finding is consistent with that observed in the cells of the proliferative zones of the hCOs. Transcriptomics scRNAseq showed that hCOs increase the proportion of cells in the outer RGC cluster, and the generation of this oRG population is essential to recapitulate proper human-specific neocortex development and to study the mechanisms of expansion *in vitro* using hCOs.

Some cells in the proliferative zone present centrioles, primary cilia, and membrane clathrin-coated pits, and the structures that serve several functions of importance for proper neurodevelopment. Centrioles are the base structure for the correct organization of microtubules during cell division, but they are also the basis for the origin of the basal body, which will give rise to the primary cilium (Preble et al., 1999). The primary cilium has an important role in cortical development and behavior of the RGCs, and its assembly/disassembly is linked to the cell cycle. It seems to play a role in the balance between self-renewal and asymmetric cleavage of RGCs (Bear and Caspary, 2024). Problems in the cilium assembly may be associated with increased proliferation of neural progenitors and macrocephaly, while its disassembly may produce the opposite effect while generating premature differentiation and cell death (Matsumoto et al., 2019). In addition, it has been observed that the primary cilium plays a role as a receptor for signals involved in cell development and differentiation (Yamamoto and Mizushima, 2021; Bear and Caspary, 2024). Clathrin-coated pits in the surrounding ciliary pocket play a role in receptor internalization (Lampe et al., 2016; Camblor-Perujo and Konon-enko, 2022).

Neurons that are produced very early in development inherit the apical-basal polarity of RGCs and migrate to the cortical plate by somal translocation (Conti and Cattaneo, 2010). Neuronal-like morphology cells surrounding the proliferative zones are observed in the hCOs. Studies of migration using labeled neurons have been carried out previously using organoid models (Birey et al., 2017, 2022).

The ultrastructure features that we observed in the neurons of the hCOs are consistent with those described previously in the brain (Garcia-Cabezas et al., 2016; Kutukova et al., 2020; Nahirney and Tremblay, 2021). The nucleus of the neuron presents several nuclear membrane indentations, perinuclear heterochromatin, and a rounded and well-defined nucleolus. The cytoplasm is usually paler with various organelles such as mitochondria, lysosomes, and Golgi complex near the nucleus, and only in some cases, glycogen granules are observed (Ong and Garey, 1991; Kutukova et al., 2020; Nahirney and Tremblay, 2021).

Once neurons have been generated and undergone radial migration, the neurite differentiation begins. In the results, we present longitudinal and transversal sections of neurites that show arranged numerous microtubules (Hoffmann et al., 2021). In neurites, we found mitochondria that could migrate using the microtubules, and as previously described, these mitochondria are small in axons, whereas dendritic mitochondria are often much longer linked with synaptic plasticity and neurite branching (Lewis et al., 2018).

The proportion of cells in the clusters of IN and EN obtained with scRNAseq increases with the time of maturation and presents functional differences at the transcriptomic level. In the neuronal clusters of hCOs that were cultured for 60 days compared to those cultured for 45 days, we found that the differentially expressed genes (DEGs) were upregulated and were enriched in pathways characteristic of neuronal functional development (regulation of synaptic transmission, neurotransmitter receptor transport…), while downregulated DEGs were linked to differentiation and developmental processes (regulation of developmental growth, the proliferation of neuronal precursor cells…). In addition, we observed changes in the expression of some genes related to neuronal cell maturation, such as gene changes for potassium ion channels (KCND2, KCNB1, and KCNQ2) and neurotransmitter receptors such as glutamate ionotropic receptor AMPA (GRIA4), glutamate ionotropic receptor NMDA Type Subunit 2A (GRIN2A), and GRIPAP1, over time in culture. These changes in gene expression throughout the development are in accordance with previously published research on the development of the human prefrontal cortex (Herring et al., 2022).

The astrocytes are the most highly represented cell type in the brain and are critical for brain function, such as supporting or providing metabolites to neurons (Guillamón-Vivancos et al., 2015). Astrocytes are in close contact with dendrites due to their participation in synapsis; for example, the perisynthesis astrocytic processes (PAPs) produce neurotransmitters (Morita, 2023). We observed in the hCOs, which are close to neurons, that cells consistent with the ultrastructure of astrocytes were reported in the literature (Cragg, 1976; Nahirney and Tremblay, 2021; Morita, 2023). The low electron density in the nucleus of the astrocyte with the presence of euchromatin and a lack of heterochromatin could be explained by the synthesis of metabolites and neurotransmitters. Astrocytes in the hCOs present an elevated number of mitochondria, and accumulations of glycogen granules are needed for the high energy rate that astrocytes provide to neurons (Cragg, 1976; Aten et al., 2022).

The presence of multiple IFs in the cytoplasm could be explained by the structural function of astrocytes (Cragg, 1976; Pekny and Wilhelmsson, 2006; Nahirney and Tremblay, 2021; Aten et al., 2022). These IFs are mainly composed of GFAP, which is the main astrocyte-associated marker (Bignami et al., 1972; Giménez Y Ribotta et al., 2000). However, astrocyte IFs are also detected to be heteropolymers with the presence of Vimentin (Pekny and Wilhelmsson, 2006; Hohmann and Dehghani, 2019). In TEM images, we also observed microtubules. Previously, it has been reported that the rat nerve at birth shows astrocytes that contain microtubules, but during maturation, microtubules disappear almost entirely from the cytoplasm and are replaced by IFs (Peters and Vaughn, 1967; Weigel et al., 2021).

The presence of neurons and astrocytes has been described in hCOs much more frequently than the presence of oligodendrocytes (Pavlinov et al., 2023). Several diseases are related to the malfunction of oligodendrocytes because they execute myelination, assisting in the transmission of the electrical impulse and providing metabolic support to the axons. However, ultrastructural studies of oligodendrocytes in human brain tissue are scarce. Oligodendrocyte precursor cells (OPCs) (Marton et al., 2019; Cristobal and Lee, 2022) express markers, such as PDGFRA, and they differentiate into pre-oligodendrocytes or pre-myelinating oligodendrocytes (pre-OLs), which can be labeled for CNPase (Fard et al., 2017). The OPCs in the hCOs exhibit an accumulation of heterochromatin attached to the nuclear membrane and in their cytoplasm, many small mitochondria, and dilated short endoplasmic reticulum (Nahirney and Tremblay, 2021; De Kleijn et al., 2023). In comparison, a study conducted by Ulloa-Navas et al. (2021) showed that the ultrastructure of the cell found in the hCOs is similar to mature human oligodendrocytes; however, we cannot see myelin sheaths close, although there is an expression of the myelin protein PLP1. We suggest that this could be due to myelin studies requiring exquisite fixation.

Microglia are the immune cells in the brain mediating inflammatory responses and are also crucial for the regulation of developmental, homeostatic, and pathological processes associated with vasculature, synapses, and myelinization of axons (Salter and Stevens, 2017). The generation of neurons, astrocytes, and oligodendrocytes are derived from common neural stem cell progenitors; however, the origin of microglia cells is mesodermal (Garaschuk and Verkhratsky, 2019) and they are present in the hCOs, which is probably due to the use of an unguided generation protocol (González-Sastre et al., 2024).

Microglia cells found in hCOs can be discerned by TEM from other cell types by their small size and characteristic heterochromatin near the nuclear envelope. As previously described, the microglia cells present a thin cytoplasm that contains a few organelles as long stretches of endoplasmic reticulum, and it could be visible in some cases in the mitochondria, Golgi saccules, and lysosomes (Savage et al., 2018). The ultrastructural feature associated with the disease is the presence of dark microglia, which display many signs of cellular stress such as dark cytoplasm, nuclear and chromatin condensation, and great dilation of their endoplasmic reticulum (St-Pierre et al., 2019).

This detailed characterization, in addition to validating the hCO model, paves the way for the study of cellular structural changes associated with neurodevelopment.

## Data availability statement

The raw data supporting the conclusions of this article will be made available by the authors, without undue reservation.

## Ethics statement

The work conducted in the present study was approved by the ISCIII Ethics Committee (Ref. CEI-PI-93_2020 and CEI-PI-76_2023) and then authorized by the Spanish National Committee of Guarantees for the Use and Derivation of Human Cells and Tissues.

## Author contributions

PM-M: Data curation, Investigation, Methodology, Software, Writing – original draft, Writing – review & editing. RC: Investigation, Methodology, Writing – review & editing. MS: Methodology, Software, Writing – review & editing. RG-S: Investigation, Methodology, Writing – review & editing. LM: Methodology, Writing – review & editing. MR: Methodology, Software, Writing – review & editing. MT: Methodology, Software, Writing – review & editing. VL-A: Conceptualization, Investigation, Software, Supervision, Writing – original draft, Writing – review & editing. IL: Conceptualization, Investigation, Methodology, Supervision, Writing – original draft, Writing – review & editing.

## Supplementary material

Supplementary Figure S1 **(A)** Uniform manifold approximation and projection (UMAP) plot from unsupervised clustering of scRNAseq of the hCOs after culturing for 45 days and 60 days. Colors represent the cell clusters annotated as excitatory neurons (EN), interneurons (IN), neurons (N), intermediate precursor cells (IPC), oligodendrocyte precursors cells (OPC), astrocytes (AS), outer radial glia and astroglia (oRG + Astroglia), apical radial glia (aRG), and proliferative radial glia (pro-RG). **(B)** Bar plot displaying the proportion of each cluster of cells for hCOs after culturing for 45 days and 60 days. A dot plot indicating the expression of cell type-specific marker genes. The dot size represents the percentage of cells expressing the gene and the color gradient from low (blue) to high (red) indicates the average relative expression. **(C)** Violin plots showing the expression for VIM, SOX2, DCX, and MAP2 for hCOs after culturing for 45 days and 60 days. **(D)** The dot plot showing the expression for the cellular cluster of marker genes of CR: Cajal-Retzius; CP: choroid plexus; FD: forebrain dorsal; FV: forebrain ventral; HY: hippocampus; and MB/HB: midbrain and hindbrain. **(E)** Representation of enriched GO terms for upregulated and downregulated DEGs of the hCOs after culturing for 60 days versus after culturing for 45 days in the excitatory neurons cluster (Done from ReviGO).

Supplementary Table S1 A list of genes in the selected enriched GO terms upregulated and downregulated from the DEGs of the hCOs after culturing for 60 days versus after culturing for 45 days in the excitatory neurons cluster.
