# Supplementary figures and images for: Human cerebral organoids: cellular composition and subcellular morphological features
Source: Front Cell Neurosci. 2024 Jun 12;18:1406839. doi: 10.3389/fncel.2024.1406839 (PMC11199856; doi:10.3389/fncel.2024.1406839)

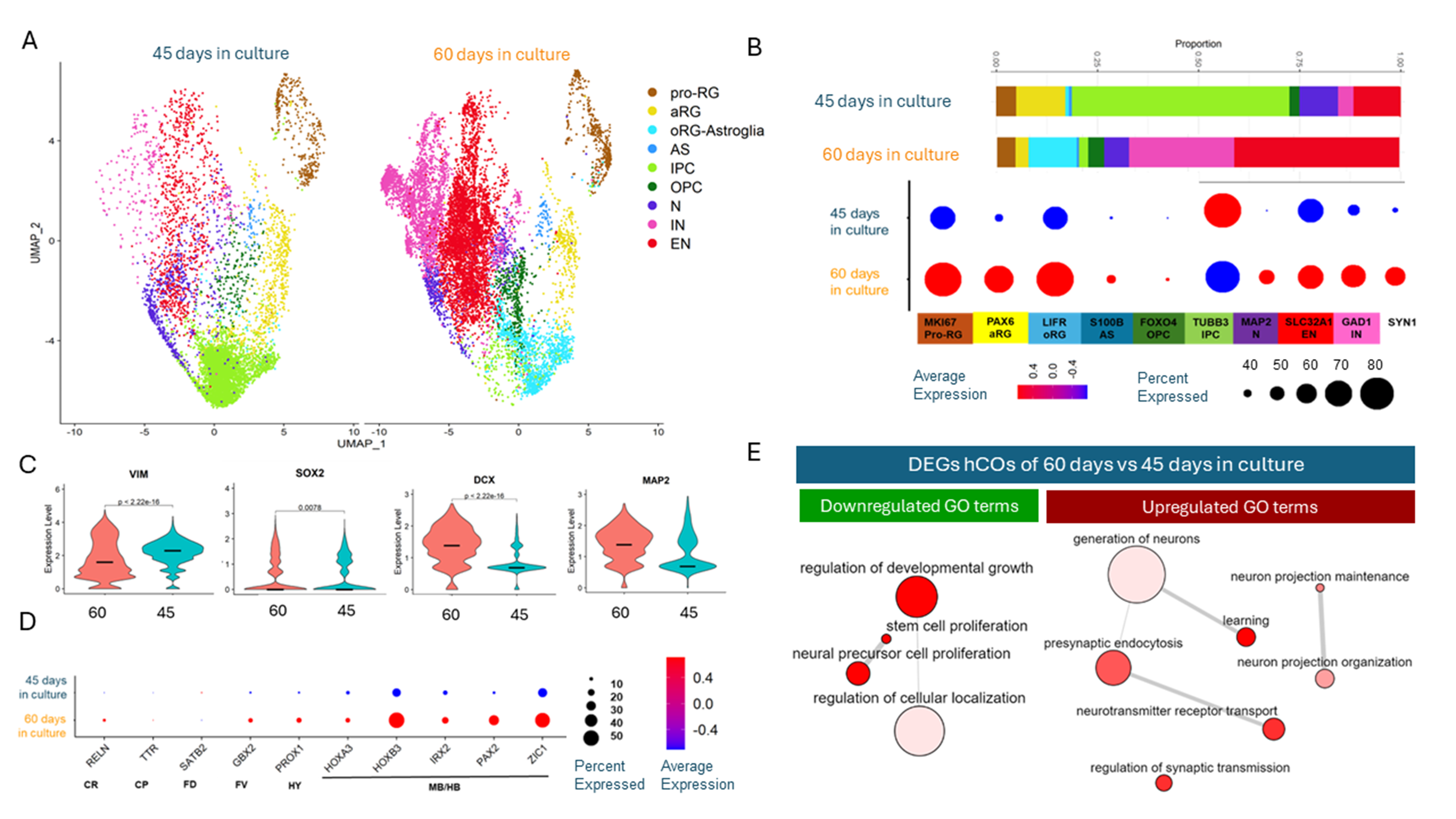

Supplement: Supplementary Figure S1 — (A) Uniform manifold approximation and projection (UMAP) plot from unsupervised clustering of scRNAseq of the hCOs after culturing for 45 days and 60 days. Colors represent the cell clusters annotated as excitatory neurons (EN), interneurons (IN), neurons (N), intermediate precursor cells (IPC), oligodendrocyte precursors cells (OPC), astrocytes (AS), outer radial glia and astroglia (oRG + Astroglia), apical radial glia (aRG), and proliferative radial glia (pro-RG). (B) Bar plot displaying the proportion of each cluster of cells for hCOs after culturing for 45 days and 60 days. A dot plot indicating the expression of cell type-specific marker genes. The dot size represents the percentage of cells expressing the gene and the color gradient from low (blue) to high (red) indicates the average relative expression. (C) Violin plots showing the expression for VIM, SOX2, DCX, and MAP2 for hCOs after culturing for 45 days and 60 days. (D) The dot plot showing the expression for the cellular cluster of marker genes of CR: Cajal-Retzius; CP: choroid plexus; FD: forebrain dorsal; FV: forebrain ventral; HY: hippocampus; and MB/HB: midbrain and hindbrain. (E) Representation of enriched GO terms for upregulated and downregulated DEGs of the hCOs after culturing for 60 days versus after culturing for 45 days in the excitatory neurons cluster (Done from ReviGO). [file Image_1.TIF]
